# No association between prenatal lead exposure and neurodevelopment during early childhood in the Japan Environment and Children’s Study

**DOI:** 10.1038/s41598-022-19509-6

**Published:** 2022-09-12

**Authors:** Hirosuke Inoue, Masafumi Sanefuji, Yuri Sonoda, Masanobu Ogawa, Norio Hamada, Masayuki Shimono, Reiko Suga, Shoji F. Nakayama, Yu Taniguchi, Koichi Kusuhara, Shouichi Ohga, Michihiro Kamijima, The Japan Environment and Children’s Study Group, Michihiro Kamijima, Shin Yamazaki, Yukihiro Ohya, Reiko Kishi, Nobuo Yaegashi, Koichi Hashimoto, Chisato Mori, Shuichi Ito, Zentaro Yamagata, Hidekuni Inadera, Takeo Nakayama, Hiroyasu Iso, Masayuki Shima, Hiroshige Nakamura, Narufumi Suganuma, Koichi Kusuhara, Takahiko Katoh

**Affiliations:** 1 Department of Pediatrics, Graduate School of Medical Sciences, Kyushu University, Fukuoka, Japan; 2 Department of Pediatrics, Faculty of Medicine, Saga University, 5-1-1 Nabashima, Saga, 849-8501 Japan; 3 Research Center for Environment and Developmental Medical Sciences, Kyushu University, Fukuoka, Japan; 4 Department of Obstetrics and Gynecology, Graduate School of Medical Sciences, Kyushu University, Fukuoka, Japan; 5 Department of Pediatrics, University of Occupational and Environmental Health, Kitakyushu, Japan; 6 Regional Center for Japan Environment and Children’s Study, University of Occupational and Environmental Health, Kitakyushu, Japan; 7 Japan Environment and Children’s Study Programme Office, National Institute for Environmental Studies, Tsukuba, Japan; 8 Department of Occupational and Environmental Health, Nagoya City University Graduate School of Medical Sciences, 1 Kawasumi, Mizuho-ku, Nagoya, Aichi 467-8601 Japan; 9 National Center for Child Health and Development, Tokyo, Japan; 10 Hokkaido University, Sapporo, Japan; 11 Tohoku University, Sendai, Japan; 12 Fukushima Medical University, Fukushima, Japan; 13 Chiba University, Chiba, Japan; 14 Yokohama City University, Yokohama, Japan; 15 University of Yamanashi, Chuo, Japan; 16 University of Toyama, Toyama, Japan; 17 Kyoto University, Kyoto, Japan; 18 Osaka University, Suita, Japan; 19 Hyogo College of Medicine, Nishinomiya, Japan; 20 Tottori University, Yonago, Japan; 21 Kochi University, Nankoku, Japan; 22 Kumamoto University, Kumamoto, Japan

**Keywords:** Environmental sciences, Neurology

## Abstract

Compared with the relatively well-investigated effects of childhood exposure to lead on neurocognitive deficits, those of prenatal exposure remain relatively inconclusive. We aimed to investigate the association between prenatal blood lead levels and neurodevelopmental delay during the first three years of life. From a prospective cohort of the Japan Environment and Children’s Study, we analyzed a total of 80,759 children. The exposure factors were prenatal lead concentrations measured from maternal whole blood in the second/third trimesters and umbilical cord blood at birth. Neurodevelopment was assessed at 6, 12, 18, 24, 30, and 36 months old using a screening tool, the Ages and Stages Questionnaires, third edition (ASQ). The outcome measures were any suspected neurodevelopmental delay (sNDD) identified via the ASQ during the first (sNDD-1Y), second (sNDD-2Y), and third (sNDD-3Y) years of life. sNDD-1Y, 2Y, and 3Y were identified in 18.0%, 16.2%, and 17.2% of children, respectively. The geometric means of blood lead concentration in this study were much lower (0.62 μg/dL in maternal blood and 0.50 μg/dL in cord blood) than previously investigated levels. Multivariable regression models revealed that there were no associations between maternal blood lead and sNDD-1Y and 2Y and between cord blood lead and sNDD-1Y, 2Y, and 3Y. Although a higher maternal blood lead was associated with a reduced risk of sNDD-3Y (adjusted relative risk: 0.84, 95% confidence interval 0.75–0.94, per 1 increase in common logarithm of lead concentration), there were no dose–response relationships in the analysis using quintiles of lead concentrations. Using a large-scale data set, the present study demonstrated no convincing evidence for an inverse association between levels of prenatal blood lead and neurodevelopment in early childhood. Longitudinal measurements of prenatal and postnatal lead levels are needed to understand the relationship between lead exposure and neurocognitive development.

## Introduction

Lead is a representative neurotoxicant that can harm children’s neurocognitive development. In 2012, the Centers for Disease Control and Prevention adopted the use of a reference blood lead concentration of ≥ 5 μg/dL as a trigger to guide clinical and public health interventions^1^. However, no threshold or safe level of lead has yet been identified or determined to even exist^2,3^. Elevated blood lead levels in children, even those below 5 μg/dL, have been associated with many developmental problems, such as intellectual deficits, diminished academic performance, attention deficits, and behavioral problems^4–6^. Childhood lead exposure may also have long-term consequences for adult mental health and personality^7,8^. Furthermore, magnetic resonance imaging studies have shown a decreased brain volume in both children and adults with lead exposure^9–11^.

Although the mechanism underlying the neurotoxicity of lead is not clearly understood, animal studies have shown that lead mimics calcium at the cellular level, interfering with neurotransmitter release and affecting neurotransmission by disturbing synaptic activity^12^. In addition, experimental animal studies suggest the vulnerability of the brain to prenatal exposure to lead^13^. However, epidemiological findings on the relationships between prenatal lead exposure and childhood neurodevelopment are inconclusive, including inverse^14–26^, null^27–34^ or even positive^16,35^ associations. These conflicting results highlight the need for the further investigation of the effect of prenatal lead exposure on child neurodevelopment.

The phase-out of leaded gasoline during the 1970s and 80 s, which was completed with prohibition of its usage in 1986, resulted in a remarkable decrease in environmental lead exposure in Japan^36^. Consequently, concentrations of lead in blood have been declining in mid/late-term pregnant women^37^. However, because lead is transferred freely across the placenta and accumulates in fetal tissues^38,39^, relatively low levels of exposure that do not greatly harm the mother might have a profound effect on the fetal brain and subsequent neurodevelopment and behavior during childhood.

The present study investigated whether or not low levels of prenatal blood lead were associated with neurodevelopment during the first three years of life using a nationwide cohort of the Japan Environment and Children’s Study (JECS). In addition, we evaluated potential sex-specific associations between prenatal lead exposure and neurodevelopment based on evidence from previous studies^14,19,20,24,26,35^. A detailed investigation of this association is valuable for determining the clinical practice guidelines for lead exposure in child-bearing women.

## Methods

### Design

The JECS is an ongoing nationwide prospective birth cohort study funded by the Ministry of the Environment, Japan. The details of the study design have been described elsewhere^40,41^. In brief, pregnant participants were registered between January 2011 and March 2014 at 15 research sites, referred to as ‘Regional Centers’, covering a wide geographical area in Japan. During the first and second/third trimesters of pregnancy, data were obtained using self-administered questionnaires. Detailed information regarding the mother and child was obtained from medical record transcripts during the first trimester, at the time of delivery, and when the child was one month old. Maternal blood samples were collected during the first and second/third trimesters, and cord blood samples were obtained at birth. After delivery, data were collected at one and six months old and twice a year thereafter via self-reported questionnaires completed by the parents or caregivers.

The JECS protocol was reviewed and approved by the Ministry of the Environment’s Institutional Review Board for Epidemiological Studies and by the Ethics Committees of all participating institutions (#100910001). This study was conducted in accordance with the principles of the Declaration of Helsinki. Written informed consent was obtained from all participants.

### Participants

**Figure 1:**
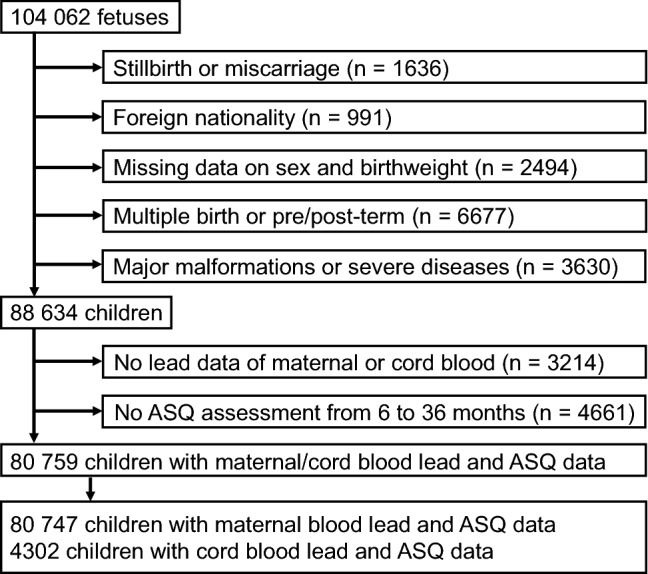
Flowchart of participant selection. *ASQ* Ages and Stages Questionnaires, third edition.

In this study, we used a dataset (jecs-ta-20190930) that was released in October 2019. The dataset contains all available data of 104,062 fetuses, linked to their mothers’ data, collected until the child was 36 months old. We selected 88,634 children with livebirth, Japanese nationality parents, available information on sex and birthweight, singleton birth, term birth (≥ 37 and < 42 gestational weeks), and no major malformations/severe diseases (Fig. 1). Of these, a total of 80,759 children were eligible for the analysis, having available data on the lead concentration in the maternal or cord blood and neurodevelopmental assessment results at any age points during the first three years of life.

### Prenatal lead exposure assessment

Prenatal exposure to lead was estimated by measuring lead concentrations in maternal blood and umbilical cord blood samples. The JECS measured lead concentrations in the maternal whole blood during the second/third trimesters from all available mothers and in the cord blood from 5% of children randomly selected among participants for whom all questionnaire responses, medical record transcripts, and biospecimens from pregnancy through 6 months old were available^42^. Ultimately, lead levels in maternal and cord blood were measured in 80,747 and 4302 children, respectively (Fig. 1).

The details of the measurement methods were described previously^37^. In brief, these concentrations were measured using inductively coupled plasma mass spectrometry on an Agilent 7700 device (Agilent Technologies, Tokyo, Japan). The concentration was provided as gravimetric units (ng/g). For the comparison with reference values and previous study reports, we converted the values to volumetric concentrations (μg/dL), using 1.0506 as the specific gravity of typical human whole blood at 37 °C^43^.

### Outcomes

To collect neurodevelopmental data from such a large number of children, the JECS used a parent-reported screening tool, the Japanese translated version of the Ages and Stages Questionnaires, third edition (ASQ), semiannually from 6 months to 3 years old. This version was prepared through a back-translation procedure and was approved by the publisher of the original English version^44^. It has been used in clinical and research settings and translated into several languages^45–47^.

The ASQ assesses five developmental domains. For each domain, six skills are described, to which parents answer “yes”, “sometimes”, or “not yet” depending on whether or not their child has demonstrated the described skill. The responses are then converted to points, with “yes” receiving 10 points; “sometimes”, 5 points; and “not yet”, 0 points. The child’s score for each developmental domain is the sum of all points received for the items under that domain and ranges from 0 to 60 points. The cut-off score for each domain is defined as two standard deviations below the mean score of large standardized samples in the United States. A child was defined as having suspected neurodevelopmental delay (sNDD) if a score was at or below the cut-off level in any developmental domain. The preliminary cut-off scores of the Japanese translated version were derived from very limited sample sizes, especially at 6, 12, and 18 months old^48^. Therefore, cut-off scores in the present study were determined using the same methodologies as in the original version, based on available data of live birth children with Japanese nationality at 6 (n = 82,413), 12 (n = 78,446), 18 (n = 73,935), 24 (n = 77,921), 30 (n = 75,761), and 36 months old (n = 77,143) in this dataset. We also conducted the same analyses using the cut-off scores of the Japanese version^48^.

Because the ASQ is a simple screening test that might overlook sNDD if administered at just one age point, we evaluated sNDD using tests conducted at two different age points. For example, a child was regarded as having sNDD during the first year of life (sNDD-1Y) when sNDD was identified at 6 and/or 12 months old. sNDD in the second (sNDD-2Y: 18 and 24 months) and third years (sNDD-3Y: 30 and 36 months) of life were defined similarly. We did not treat the ASQ score as a continuous variable because the distributions of the score at several ages were highly skewed to the left, showing a ceiling effect.

### Statistical analyses

We used quasi-Poisson regression models to estimate the risks of sNDD in association with lead levels of the maternal and cord blood. As an independent variable, these lead concentrations were analyzed in two ways. First, the concentrations were transformed into common logarithmic values (log_10_) because the distributions of these levels were highly skewed to the right^37^. Second, the levels were categorized into concentration quintiles to examine whether or not the exposure and outcome showed a dose–response relationship. The covariates included in the regression models were (i) sex, (ii) gestational age at maternal blood sampling, (iii) gestational age at birth, (iv) birthweight, (v) mother’s age, (vi) maternal smoking during pregnancy, as recorded in the first trimester, (vii) maternal education level (completed junior high school, completed high school/technical junior college, and completed bachelor’s degree), (viii) paternal education level, (ix) annual household income (< 4,000,000; 4,000,000–5,999,999; ≥ 6,000,000 JPY), (x) any breastfeeding continued during the first 6 months of life, and (xi) home speech stimulation at 1 month old (whether or not a mother talked to her baby habitually). The “home speech stimulation” covariate was used instead of the Home Observation for Measurement of the Environment scale^49^, which was not employed in the JECS protocol. We also examined the associations separately by sex.

All statistical analyses were performed using the R software program, version 4.0.3 (The R Core Team, Vienna, Austria). The results were reported with crude and adjusted relative risks (RRs) with 95% confidence intervals (CIs).

## Results

Table 1 shows the baseline characteristics of the 80,759 participants whose ASQ data were available. Geometric means (95% CI) of lead levels in maternal and cord blood were 0.621 μg/dL (0.619–0.622) and 0.502 μg/dL (0.497–0.508), respectively. The log-transformed lead concentrations were highly correlated between maternal and cord blood (Pearson correlation coefficient = 0.730). sNDD-1Y, 2Y, and 3Y were observed in 18.0%, 16.2%, and 17.2% of children, respectively; when using the cut-off scores of the Japanese version^48^, the prevalences were 25.7%, 16.6%, and 18.9%, respectively.

**Table 1:** Baseline characteristics of the children for the analysis.

	80,759 children	[Missing]
**Boy, n (%)**	41,190 (51.0)	0
**Gestational age, mean (SD), weeks**	39.5 (1.1)	0
**Birthweight, mean (SD), g**	3063 (366)	0
**Maternal age, mean (SD), years**	31.2 (5.0)	4
**Maternal smoking during pregnancy, n (%)**	13 801 (17.3)	957
**Maternal education, n (%)**		891
Completed junior high school	3506 (4.4)	
Completed High school/technical junior college	59,015 (73.9)	
Completed bachelor's degree	17,347 (21.7)	
**Paternal education, n (%)**		1344
Completed junior high school	5530 (7.0)	
Completed High school/technical junior college	47,313 (59.6)	
Completed bachelor's degree	26,572 (33.5)	
**Household income, n (%)**		6034
Low (< 4,000,000 JPY)	29,768 (39.8)	
Middle (4,000,000–5,999,999 JPY)	24,883 (33.3)	
High (≥ 6,000,000 JPY)	20,074 (26.9)	
**Continuous breastfeeding until 6 months, n (%)**	63,994 (81.5)	2242
**Home speech stimulation at 1 month, n (%)**	65,091 (81.3)	730
**Lead, geometric mean (95% CI), μg/dL**		
Maternal blood	0.621 (0.619–0.622)	12
Cord blood	0.502 (0.497–0.508)	76,457

*CI* confidence interval, *JPY* Japanese yen, *SD* standard deviation.

Multivariable regression models were used to estimate the RRs for sNDD-1Y, 2Y, and 3Y in association with lead concentrations of the maternal and cord blood. When using the logarithmic lead concentrations as an exposure factor, crude models revealed no associations of maternal or cord blood lead with sNDD in any periods (Table 2a). Although the results were largely similar after adjusting for covariates, a higher maternal blood lead was associated with reduced risks of sNDD-3Y (adjusted RR 0.84, 95% CI 0.75–0.94, per 1 increase in logarithmic lead concentration). When using the quintiles of lead concentration in the maternal blood, crude and adjusted models showed reduced risks of sNDD at several higher quintiles compared with the lowest quintile (Table 2b). However, these associations did not show an obvious dose–response relationship. With regard to cord blood, there were no significant associations between lead levels and sNDD. In sex-stratified analyses using the log-transformation and the quintiles of lead concentration, there were no evident differences between sexes (Table 3). When using the cut-off scores of the Japanese version^48^, regression analyses produced similar results (Supplementary Tables S1, S2). To address the multiple testing problem for the three periods of sNDD (Tables 2, 3), we also calculated 99% CIs of the lead exposures, which did not change the conclusions (Supplementary Tables S3, S4, respectively).

**Table 2:** Association between prenatal lead exposure and sNDD.

	sNDD-1Y		sNDD-2Y		sNDD-3Y	
	Crude	Adjusted	Crude	Adjusted	Crude	Adjusted
**a. Log-transformed lead (log**_**10**_**)**						
Maternal blood	0.97 (0.87–1.06)	0.92 (0.83–1.02)	0.98 (0.88–1.09)	0.89 (0.80–1.00)	0.98 (0.88–1.09)	**0.84 (0.75–0.94)**
Cord blood	1.08 (0.74–1.57)	1.08 (0.72–1.60)	1.43 (0.95–2.15)	1.31 (0.85–2.00)	0.98 (0.67–1.42)	0.88 (0.59–1.31)
**b. Quintiles of lead (µg/dL)**						
**Maternal blood**						
Q1 (< 0.468)	1 (reference)	1 (reference)	1 (reference)	1 (reference)	1 (reference)	1 (reference)
Q2 (0.468–0.564)	**0.94 (0.89–0.98)**	**0.94 (0.90–0.99)**	0.95 (0.90–1.00)	0.95 (0.90–1.01)	0.96 (0.92–1.01)	0.95 (0.90–1.00)
Q3 (0.564–0.666)	**0.94 (0.90–0.98)**	0.95 (0.91–1.00)	0.96 (0.91–1.01)	0.96 (0.91–1.01)	**0.93 (0.88–0.98)**	**0.90 (0.86–0.95)**
Q4 (0.666–0.813)	**0.92 (0.88–0.97)**	**0.93 (0.88–0.97)**	0.96 (0.92–1.02)	0.96 (0.91–1.01)	**0.95 (0.90–1.00)**	**0.93 (0.88–0.98)**
Q5 (> 0.813)	0.98 (0.93–1.02)	**0.95 (0.90–1.00)**	0.99 (0.94–1.04)	**0.95 (0.89–1.00)**	0.99 (0.94–1.04)	**0.92 (0.88–0.98)**
**Cord blood lead**						
Q1 (< 0.373)	1 (reference)	1 (reference)	1 (reference)	1 (reference)	1 (reference)	1 (reference)
Q2 (0.373–0.451)	1.00 (0.82–1.22)	1.04 (0.84–1.28)	1.10 (0.88–1.36)	1.10 (0.88–1.37)	0.98 (0.81–1.18)	1.01 (0.82–1.23)
Q3 (0.451–0.540)	0.91 (0.74–1.12)	0.93 (0.75–1.15)	0.95 (0.76–1.20)	0.92 (0.73–1.17)	1.01 (0.84–1.22)	1.02 (0.83–1.24)
Q4 (0.540–0.676)	1.11 (0.91–1.34)	1.12 (0.92–1.37)	1.16 (0.94–1.44)	1.07 (0.86–1.34)	1.01 (0.83–1.22)	1.02 (0.84–1.25)
Q5 (> 0.676)	1.01 (0.83–1.23)	0.97 (0.78–1.19)	1.19 (0.96–1.47)	1.07 (0.85–1.34)	0.95 (0.79–1.15)	0.88 (0.71–1.08)

Values are presented as relative risks (95% confidence intervals) adjusted for sex, gestational ages at maternal blood sampling and at birth, birthweight, mother's age, maternal smoking, maternal and paternal education, household income, continuous breastfeeding until 6 months, and home speech stimulation at 1 month. Bold text represents statistical significance (p < 0.05).

*sNDD* suspected neurodevelopmental delay, *Q* quintile.

**Table 3:** Sex-stratified association between prenatal lead exposure and sNDD.

	sNDD-1Y		sNDD-2Y		sNDD-3Y	
	Crude	Adjusted	Crude	Adjusted	Crude	Adjusted
**a. Log-transformed lead (log**_**10**_**)**						
**Maternal blood**						
Boy	0.97 (0.85–1.11)	0.93 (0.81–1.07)	0.94 (0.81–1.07)	0.88 (0.76–1.02)	0.91 (0.80–1.03)	**0.85 (0.75–0.98)**
Girl	0.91 (0.79–1.06)	0.90 (0.77–1.06)	0.95 (0.80–1.12)	0.91 (0.76–1.09)	0.88 (0.73–1.07)	0.82 (0.67–1.00)
**Cord blood**						
Boy	1.08 (0.64–1.78)	1.06 (0.62–1.80)	1.25 (0.74–2.10)	1.21 (0.70–2.08)	0.77 (0.50–1.18)	0.74 (0.47–1.17)
Girl	1.04 (0.59–1.83)	1.13 (0.62–2.02)	1.60 (0.83–3.06)	1.53 (0.76–3.05)	1.28 (0.64–2.52)	1.35 (0.64–2.82)
**b. Quintiles of lead (µg/dL)**						
**Maternal blood**						
**Boy**						
Q1 (< 0.468)	1 (reference)	1 (reference)	1 (reference)	1 (reference)	1 (reference)	1 (reference)
Q2 (0.468–0.564)	**0.92 (0.86–0.98)**	**0.92 (0.86–0.98)**	0.97 (0.90–1.03)	0.96 (0.89–1.03)	0.96 (0.90–1.02)	0.95 (0.89–1.01)
Q3 (0.564–0.666)	**0.94 (0.88–1.00)**	0.94 (0.88–1.01)	0.94 (0.87–1.00)	**0.93 (0.86–1.00)**	**0.91 (0.86–0.97)**	**0.90 (0.85–0.96)**
Q4 (0.666–0.813)	**0.92 (0.86–0.98)**	**0.92 (0.86–0.99)**	0.95 (0.89–1.01)	0.95 (0.89–1.02)	**0.93 (0.87–0.98)**	**0.92 (0.87–0.98)**
Q5 (> 0.813)	0.97 (0.91–1.03)	0.95 (0.89–1.02)	0.97 (0.91–1.04)	0.94 (0.87–1.01)	0.96 (0.90–1.02)	**0.94 (0.88–1.00)**
**Girl**						
Q1 (< 0.468)	1 (reference)	1 (reference)	1 (reference)	1 (reference)	1 (reference)	1 (reference)
Q2 (0.468–0.564)	0.96 (0.89–1.03)	0.97 (0.90–1.04)	**0.92 (0.85–1.00)**	0.94 (0.87–1.03)	0.94 (0.86–1.03)	0.95 (0.87–1.05)
Q3 (0.564–0.666)	**0.93 (0.87–1.00)**	0.97 (0.90–1.04)	0.98 (0.91–1.06)	1.01 (0.93–1.10)	**0.91 (0.84–1.00)**	**0.90 (0.82–1.00)**
Q4 (0.666–0.813)	**0.91 (0.85–0.98)**	**0.93 (0.86–1.00)**	0.96 (0.89–1.04)	0.97 (0.89–1.06)	**0.92 (0.84–1.01)**	0.95 (0.86–1.04)
Q5 (> 0.813)	0.96 (0.90–1.03)	0.95 (0.88–1.02)	0.97 (0.89–1.05)	0.96 (0.88–1.05)	0.95 (0.87–1.04)	**0.90 (0.81–0.99)**
**Cord blood lead**						
**Boy**						
Q1 (< 0.373)	1 (reference)	1 (reference)	1 (reference)	1 (reference)	1 (reference)	1 (reference)
Q2 (0.373–0.451)	0.93 (0.71–1.22)	0.96 (0.72–1.28)	1.02 (0.77–1.35)	0.98 (0.73–1.31)	0.98 (0.79–1.23)	1.04 (0.82–1.31)
Q3 (0.451–0.540)	0.92 (0.70–1.21)	0.94 (0.71–1.25)	0.90 (0.67–1.20)	0.85 (0.63–1.16)	1.02 (0.82–1.26)	1.03 (0.82–1.31)
Q4 (0.540–0.676)	1.14 (0.89–1.46)	1.15 (0.88–1.50)	1.16 (0.89–1.51)	1.09 (0.83–1.45)	0.91 (0.73–1.14)	0.98 (0.77–1.24)
Q5 (> 0.676)	0.93 (0.71–1.21)	0.89 (0.66–1.19)	1.11 (0.85–1.45)	1.04 (0.78–1.39)	0.92 (0.74–1.15)	0.88 (0.69–1.12)
**Girl**						
Q1 (< 0.373)	1 (reference)	1 (reference)	1 (reference)	1 (reference)	1 (reference)	1 (reference)
Q2 (0.373–0.451)	1.08 (0.81–1.44)	1.13 (0.84–1.52)	1.23 (0.88–1.73)	1.28 (0.90–1.81)	1.00 (0.71–1.41)	0.98 (0.67–1.42)
Q3 (0.451–0.540)	0.90 (0.66–1.22)	0.92 (0.66–1.27)	1.04 (0.73–1.49)	1.03 (0.71–1.50)	0.98 (0.69–1.39)	0.98 (0.67–1.42)
Q4 (0.540–0.676)	1.04 (0.77–1.40)	1.05 (0.77–1.44)	1.13 (0.79–1.61)	1.03 (0.70–1.51)	1.14 (0.81–1.60)	1.09 (0.76–1.57)
Q5 (> 0.676)	1.11 (0.83–1.48)	1.12 (0.82–1.53)	1.29 (0.91–1.82)	1.12 (0.78–1.62)	0.95 (0.66–1.36)	0.89 (0.59–1.32)

Values are presented as relative risks (95% confidence intervals) adjusted for sex, gestational ages at maternal blood sampling and at birth, birthweight, mother's age, maternal smoking, maternal and paternal education, household income, continuous breastfeeding until 6 months, and home speech stimulation at 1 month. Bold text represents statistical significance (p < 0.05).

*sNDD* suspected neurodevelopmental delay, *Q* quintile.

## Discussion

Using a nationwide birth cohort in Japan, the present study investigated the association between prenatal lead levels and neurodevelopment during the first 3 years of life. The lead levels were very low in both the maternal and cord blood. In multivariable regression analyses using log-transformed lead concentration, no associations were observed in the majority of the combinations between maternal/cord lead levels and sNDD for the 3-year period. However, a higher level of maternal blood lead was associated with a reduced risk of child’s sNDD in the third years of life. In the analyses using the quintiles of lead concentration, several higher quintiles versus the lowest quintile of maternal lead were inversely associated with the risk of sNDD at the three age points, but these associations did not show an obvious dose–response relationship. Ultimately, this study did not demonstrate any definite associations between very low levels of prenatal lead and neurodevelopment during early childhood.

The most important clinically relevant finding was that the prenatal lead exposure was very low in our study. We analyzed survey data with a large sample size and found that the prenatal exposure to such low levels of lead was not associated with the risk of sNDD in the child. Several studies demonstrated that child neurodevelopment was inversely related to prenatal lead exposure, measured via maternal and/or cord blood lead levels^14–26^, although others reported null^27–34^ or even positive associations^16,35^. Comparing these studies is difficult because there are variations in the timing of blood sampling during the prenatal period, the method and age of the cognitive function tests, the number of participants, and the statistical modeling techniques. Importantly, the maternal blood lead concentrations in our study were much lower than those in the majority of these earlier studies. Such low lead levels might have had negligible adverse effects on the child’s neurodevelopment and thus yielded null associations in our study.

However, our null results may not be solely attributable to very low levels of prenatal lead exposure. A few studies still reported inverse associations between prenatal lead levels and the neurocognitive function when the levels were comparable to those in our cohort^14,17,26^. A child’s cognitive function is reportedly better predicted by concurrent blood lead levels than past levels^3,50,51^, even at very low levels of exposure^52,53^. These findings suggest that exposure to lead at very early developmental stages has less impact on the brain than that at later stages. The low significance of the early period may explain the inconsistent association between prenatal lead exposure and children’s neurodevelopment. Although very low levels of lead might damage the fetal brain, the damaging effects might be obscured by the effects of postnatal exposure to chemicals, including lead and other environmental factors, that a child experiences after birth. Lead-induced brain injury may thus be plastic and reversible when the exposure is very low during the fetal period.

The less consistent associations with neurodevelopment of prenatal than postnatal lead exposure might be further explained by variable correlations between prenatal and postnatal lead concentrations. Usually, the postnatal lead concentration would be highly correlated with the prenatal one when the living environment is relatively stable. However, such correlations may be diminished in some circumstances. For example, postnatal lead levels may decrease below those estimated from prenatal lead levels if the child’s family moves to a low-lead-polluted location or renovates their home using leadless materials. Conversely, postnatal levels may be higher than expected if the child strongly adopts mouthing behavior, which would facilitate the ingestion of lead-contaminated house dust. Postnatal lead levels are associated inversely with neurocognitive functions^3,4^. When prenatal and postnatal lead levels are highly correlated, the prenatal levels may be a proxy for the postnatal levels and thus may show an inverse association with the neurocognitive function. In contrast, when there is less correlation between these levels, an association between prenatal lead exposure and the cognitive function may not be observed. Although only prenatal lead levels were measured in the JECS cohort at present, childhood lead levels will be available in the future, enabling the determination of the sensitive periods and cumulative effects of lead exposure on children’s neurodevelopment.

Several limitations associated with the present study warrant mention. First, the identified sNDD might be somewhat equivocal, as it relied solely on the parent-reported screening test of ASQ using self-administered questionnaires. Furthermore, it had to be assessed dichotomously (presence or absence of sNDD), which may miss subtle differences in neurocognitive functions, possibly resulting in less statistical power. Second, although our statistical models accounted for several covariates, including parental education and household income, there may have been more important factors that might have influenced the results. In particular, the models did not include maternal intelligence, which greatly determines neurocognitive development in offspring. Furthermore, other neurotoxicants that may affect neurocognitive outcomes when co-exposed to lead, such as manganese, which has been reported to enhance neurotoxicity upon co-exposure^54,55^, were not examined. Third, with regard to cord blood, because the lead concentrations were measured only in a very limited portion of children, the results had relatively low certainty. Finally, the regression models indicated several associations between a higher maternal lead level and a reduced risk of sNDD during the tested periods, whereas a dose–response relationship was not noted. Such associations might be due to an artifactual statistical effect known as the ‘reversal paradox’^56^.

## Conclusion

In the current study, very low levels of prenatal blood lead did not obviously have an inverse association with neurodevelopment during the first 3 years of life. The JECS plans to conduct lead measurements and face-to-face cognitive tests repeatedly during childhood. These efforts will precisely determine the optimal measurement times and acceptable levels of lead exposure from the fetal period through childhood.

## Supplementary Information

Supplementary Information.

## Data Availability

Data are unsuitable for public deposition due to ethical restrictions and the legal framework of Japan. It is prohibited by the Act on the Protection of Personal Information (Act No. 57 of 30 May 2003, amendment on 9 September 2015) to publicly deposit data containing personal information. The Ethical Guidelines for Medical and Health Research Involving Human Subjects enforced by the Japan Ministry of Education, Culture, Sports, Science and Technology and the Ministry of Health, Labour and Welfare also restrict the open sharing of epidemiologic data. All inquiries about access to data should be sent to: jecs-en@nies.go.jp. The person responsible for handling enquiries sent to this e-mail address is Dr. Shoji F. Nakayama, JECS Programme Office, National Institute for Environmental Studies.

## Abbreviations

ASQ: Ages and Stages Questionnaires, third edition
CI: Confidence interval
JECS: Japan Environment and Children’s Study
sNDD: Suspected neurodevelopmental delay
RR: Relative risk
