# Supplementary material for: No association between prenatal lead exposure and neurodevelopment during early childhood in the Japan Environment and Children’s Study
Source: Sci Rep. 2022 Sep 12;12:15305. doi: 10.1038/s41598-022-19509-6 (PMC9468004; doi:10.1038/s41598-022-19509-6)
Supplement: Supplementary file 1 — Supplementary Information. [file 41598_2022_19509_MOESM1_ESM.docx]

*Supplementary information for*

**No association between prenatal lead and neurodevelopment during early childhood in the Japan Environment and Children’s Study**

Hirosuke Inoue, Masafumi Sanefuji, Yuri Sonoda, Masanobu Ogawa, Norio Hamada, Masayuki Shimono, Reiko Suga, Shoji F. Nakayama, Yu Taniguchi, Koichi Kusuhara, Shouichi Ohga, Michihiro Kamijima, The Japan Environment and Children’s Study Group

| **Supplementary Table S1.** Association between prenatal lead exposure and sNDD using cut-off scores of Japanese ASQ | | | | | | | | |
| --- | --- | --- | --- | --- | --- | --- | --- | --- |
| **a.** Log-transformed   Lead (log_10_) | sNDD-1Y | |  | sNDD-2Y | |  | sNDD-3Y | |
|  | crude | adjusted |  | crude | adjusted |  | crude | adjusted |
| Maternal blood | 0.92 (0.85-0.99) | 0.87 (0.80-0.94) |  | 0.95 (0.85-1.06) | **0.88 (0.78-0.98)** |  | 0.96 (0.87-1.06) | **0.83 (0.75-0.92)** |
| Cord blood | 1.07 (0.79-1.45) | 1.04 (0.75-1.43) |  | 1.38 (0.92-2.05) | 1.30 (0.85-1.98) |  | 1.14 (0.80-1.61) | 1.02 (0.71-1.48) |
|  |  |  |  |  |  |  |  |  |
| **b.** Quintiles of   Lead (µg/dL) | sNDD-1Y | |  | sNDD-2Y | |  | sNDD-3Y | |
|  | crude | adjusted |  | crude | adjusted |  | crude | adjusted |
| Maternal blood |  |  |  |  |  |  |  |  |
| Q1 (< 0.468) | 1 (reference) | 1 (reference) |  | 1 (reference) | 1 (reference) |  | 1 (reference) | 1 (reference) |
| Q2 (0.468–0.564) | **0.95 (0.92–0.99)** | **0.95 (0.92–0.99)** |  | 0.95 (0.91–1.00) | 0.95 (0.90–1.00) |  | **0.95 (0.91–1.00)** | **0.93 (0.89–0.98)** |
| Q3 (0.564–0.666) | **0.96 (0.92–0.99)** | 0.97 (0.93–1.01) |  | 0.96 (0.91–1.01) | 0.95 (0.90–1.01) |  | **0.93 (0.89–0.97)** | **0.90 (0.86–0.95)** |
| Q4 (0.666–0.813) | **0.92 (0.88–0.95)** | **0.92 (0.88–0.95)** |  | **0.95 (0.90–1.00)** | **0.94 (0.89–1.00)** |  | **0.94 (0.90–0.99)** | **0.92 (0.87–0.97)** |
| Q5 (> 0.813) | 0.97 (0.93–1.01) | **0.94 (0.90–0.98)** |  | 0.98 (0.93–1.03) | **0.94 (0.89–1.00)** |  | 0.98 (0.94–1.03) | **0.92 (0.87–0.97)** |
| Cord blood lead |  |  |  |  |  |  |  |  |
| Q1 (< 0.373) | 1 (reference) | 1 (reference) |  | 1 (reference) | 1 (reference) |  | 1 (reference) | 1 (reference) |
| Q2 (0.373–0.451) | 1.06 (0.91–1.25) | 1.11 (0.94–1.31) |  | 1.12 (0.90–1.38) | 1.13 (0.90–1.40) |  | 0.96 (0.80–1.15) | 0.96 (0.79–1.17) |
| Q3 (0.451–0.540) | 1.03 (0.88–1.22) | 1.07 (0.90–1.26) |  | 1.01 (0.81–1.26) | 0.98 (0.78–1.24) |  | 1.02 (0.85–1.22) | 1.01 (0.84–1.22) |
| Q4 (0.540–0.676) | 1.06 (0.90–1.24) | 1.08 (0.91–1.28) |  | 1.13 (0.91–1.40) | 1.05 (0.84–1.32) |  | 1.06 (0.89–1.26) | 1.04 (0.87–1.26) |
| Q5 (> 0.676) | 1.05 (0.89–1.23) | 1.01 (0.85–1.21) |  | 1.20 (0.98–1.48) | 1.10 (0.88–1.38) |  | 0.98 (0.82–1.18) | 0.90 (0.74–1.10) |
| Values are presented as relative risks (95% confidence intervals) adjusted for sex, gestational ages at maternal blood sampling and at birth, birthweight, mother's age, maternal smoking, maternal and paternal education, household income, continuous breastfeeding until 6 months, and home speech stimulation at 1 month. Bold text represents statistical significance (p < 0.05).  *ASQ* Ages & Stages Questionnaires, third edition, *sNDD* suspected neurodevelopmental delay, *Q* quintile | | | | | | | | |

| **Supplementary Table S2.** Sex-stratified association between prenatal lead exposure and sNDD using cut-off scores of Japanese ASQ | | | | | | | | |
| --- | --- | --- | --- | --- | --- | --- | --- | --- |
| **a.** Log-transformed   Lead (log_10_) | sNDD-1Y | |  | sNDD-2Y | |  | sNDD-3Y | |
|  | crude | adjusted |  | crude | adjusted |  | crude | adjusted |
| Maternal blood |  |  |  |  |  |  |  |  |
| Boy | 0.96 (0.86-1.07) | 0.91 (0.82-1.02) |  | 0.91 (0.79-1.05) | **0.86 (0.74-0.99)** |  | 0.90 (0.80-1.02) | **0.85 (0.75-0.96)** |
| Girl | **0.86 (0.76-0.96)** | 0.82 (0.72-0.92) |  | 0.92 (0.78-1.09) | 0.89 (0.75-1.07) |  | 0.85 (0.71-1.02) | **0.79 (0.65-0.96)** |
| Cord blood |  |  |  |  |  |  |  |  |
| Boy | 1.03 (0.68-1.57) | 0.94 (0.60-1.46) |  | 1.20 (0.71-2.00) | 1.19 (0.68-2.05) |  | 0.87 (0.58-1.30) | 0.83 (0.54-1.27) |
| Girl | 1.09 (0.69-1.70) | 1.17 (0.73-1.88) |  | 1.56 (0.82-2.92) | 1.52 (0.77-2.98) |  | 1.56 (0.82-2.97) | 1.68 (0.83-3.35) |
|  |  |  |  |  |  |  |  |  |
| **b.** Quintiles of   Lead (µg/dL) | sNDD-1Y | |  | sNDD-2Y | |  | sNDD-3Y | |
|  | crude | adjusted |  | crude | adjusted |  | crude | adjusted |
| Maternal blood |  |  |  |  |  |  |  |  |
| Boy |  |  |  |  |  |  |  |  |
| Q1 (< 0.468) | 1 (reference) | 1 (reference) |  | 1 (reference) | 1 (reference) |  | 1 (reference) | 1 (reference) |
| Q2 (0.468–0.564) | **0.92 (0.87-0.97)** | **0.92 (0.87-0.97)** |  | 0.98 (0.91-1.04) | 0.97 (0.90-1.04) |  | 0.95 (0.90-1.00) | **0.93 (0.88-0.99)** |
| Q3 (0.564–0.666) | 0.96 (0.91-1.01) | 0.96 (0.91-1.02) |  | **0.92 (0.86-0.99)** | **0.91 (0.85-0.98)** |  | **0.92 (0.87-0.97)** | **0.91 (0.86-0.96)** |
| Q4 (0.666–0.813) | **0.91 (0.87-0.96)** | **0.91 (0.86-0.96)** |  | **0.93 (0.87-0.99)** | **0.93 (0.87-1.00)** |  | **0.92 (0.87-0.98)** | **0.91 (0.86-0.97)** |
| Q5 (> 0.813) | 0.97 (0.92-1.02) | 0.95 (0.90-1.00) |  | 0.97 (0.91-1.03) | 0.94 (0.87-1.00) |  | 0.96 (0.91-1.01) | **0.93 (0.88-0.99)** |
| Girl |  |  |  |  |  |  |  |  |
| Q1 (< 0.468) | 1 (reference) | 1 (reference) |  | 1 (reference) | 1 (reference) |  | 1 (reference) | 1 (reference) |
| Q2 (0.468–0.564) | 0.98 (0.93-1.04) | 0.99 (0.94-1.05) |  | **0.91 (0.84-0.99)** | 0.93 (0.86-1.01) |  | 0.92 (0.85-1.00) | 0.93 (0.85-1.02) |
| Q3 (0.564–0.666) | 0.95 (0.90-1.00) | 0.97 (0.92-1.03) |  | 0.98 (0.91-1.06) | 1.01 (0.93-1.10) |  | **0.90 (0.83-0.98)** | **0.90 (0.82-0.98)** |
| Q4 (0.666–0.813) | **0.92 (0.87-0.97)** | **0.92 (0.87-0.98)** |  | 0.96 (0.88-1.03) | 0.97 (0.89-1.05) |  | **0.91 (0.84-0.99)** | 0.93 (0.85-1.02) |
| Q5 (> 0.813) | 0.96 (0.91-1.01) | **0.92 (0.87-0.98)** |  | 0.96 (0.89-1.04) | 0.95 (0.87-1.04) |  | 0.94 (0.86-1.02) | **0.89 (0.81-0.98)** |
| Cord blood lead |  |  |  |  |  |  |  |  |
| Boy |  |  |  |  |  |  |  |  |
| Q1 (< 0.373) | 1 (reference) | 1 (reference) |  | 1 (reference) | 1 (reference) |  | 1 (reference) | 1 (reference) |
| Q2 (0.373–0.451) | 1.04 (0.83-1.29) | 1.06 (0.85-1.34) |  | 1.04 (0.79-1.38) | 1.00 (0.75-1.34) |  | 1.00 (0.81-1.23) | 1.01 (0.81-1.27) |
| Q3 (0.451–0.540) | 0.99 (0.79-1.24) | 0.99 (0.78-1.25) |  | 0.94 (0.70-1.25) | 0.89 (0.66-1.21) |  | 1.05 (0.85-1.29) | 1.06 (0.85-1.32) |
| Q4 (0.540–0.676) | 1.02 (0.82-1.27) | 0.99 (0.79-1.25) |  | 1.12 (0.86-1.46) | 1.06 (0.80-1.40) |  | 0.97 (0.79-1.20) | 1.01 (0.81-1.26) |
| Q5 (> 0.676) | 1.05 (0.85-1.30) | 1.01 (0.80-1.27) |  | 1.12 (0.86-1.47) | 1.08 (0.81-1.44) |  | 0.96 (0.78-1.18) | 0.90 (0.72-1.14) |
| Girl |  |  |  |  |  |  |  |  |
| Q1 (< 0.373) | 1 (reference) | 1 (reference) |  | 1 (reference) | 1 (reference) |  | 1 (reference) | 1 (reference) |
| Q2 (0.373–0.451) | 1.09 (0.87-1.38) | 1.16 (0.91-1.48) |  | 1.24 (0.89-1.73) | 1.30 (0.93-1.82) |  | 0.92 (0.66-1.28) | 0.89 (0.62-1.28) |
| Q3 (0.451–0.540) | 1.08 (0.85-1.37) | 1.13 (0.88-1.44) |  | 1.12 (0.79-1.58) | 1.11 (0.77-1.59) |  | 0.95 (0.68-1.33) | 0.91 (0.64-1.30) |
| Q4 (0.540–0.676) | 1.09 (0.86-1.39) | 1.15 (0.89-1.48) |  | 1.10 (0.78-1.57) | 1.02 (0.70-1.49) |  | 1.15 (0.84-1.59) | 1.10 (0.78-1.54) |
| Q5 (> 0.676) | 1.03 (0.81-1.31) | 1.05 (0.81-1.37) |  | 1.30 (0.93-1.82) | 1.15 (0.81-1.65) |  | 0.96 (0.69-1.35) | 0.91 (0.63-1.31) |
| Values are presented as relative risks (95% confidence intervals) adjusted for sex, gestational ages at maternal blood sampling and at birth, birthweight, mother's age, maternal smoking, maternal and paternal education, household income, continuous breastfeeding until 6 months, and home speech stimulation at 1 month. Bold text represents statistical significance (p < 0.05).  *ASQ* Ages & Stages Questionnaires, third edition, *sNDD* suspected neurodevelopmental delay, *Q* quintile | | | | | | | | |

| **Supplementary Table S3.** Association between prenatal lead and sNDD using 99% confidence intervals of lead exposure | | | | | | | | |
| --- | --- | --- | --- | --- | --- | --- | --- | --- |
| **a.** Log-transformed   Lead (log_10_) | sNDD-1Y | |  | sNDD-2Y | |  | sNDD-3Y | |
|  | crude | adjusted |  | crude | adjusted |  | crude | adjusted |
| Maternal blood | 0.97 (0.85-1.10) | 0.92 (0.80-1.05) |  | 0.98 (0.85-1.13) | 0.89 (0.77-1.04) |  | 0.98 (0.85-1.13) | **0.84 (0.73-0.98)** |
| Cord blood | 1.08 (0.65-1.77) | 1.08 (0.64-1.81) |  | 1.43 (0.84-2.44) | 1.31 (0.74-2.28) |  | 0.98 (0.60-1.59) | 0.88 (0.52-1.47) |
|  |  |  |  |  |  |  |  |  |
| **b.** Quintiles of   Lead (µg/dL) | sNDD-1Y | |  | sNDD-2Y | |  | sNDD-3Y | |
|  | crude | adjusted |  | crude | adjusted |  | crude | adjusted |
| Maternal blood |  |  |  |  |  |  |  |  |
| Q1 (< 0.468) | 1 (reference) | 1 (reference) |  | 1 (reference) | 1 (reference) |  | 1 (reference) | 1 (reference) |
| Q2 (0.468–0.564) | **0.94 (0.88–1.00)** | 0.94 (0.88–1.00) |  | 0.95 (0.89–1.02) | 0.95 (0.89–1.02) |  | 0.96 (0.90–1.03) | 0.95 (0.89–1.02) |
| Q3 (0.564–0.666) | **0.94 (0.88–1.00)** | 0.95 (0.89–1.02) |  | 0.96 (0.90–1.03) | 0.96 (0.89–1.03) |  | **0.93 (0.87–0.99)** | **0.90 (0.84–0.97)** |
| Q4 (0.666–0.813) | **0.92 (0.87–0.98)** | **0.93 (0.87–0.99)** |  | 0.96 (0.90–1.03) | 0.96 (0.89–1.03) |  | 0.95 (0.89–1.01) | **0.93 (0.87–1.00)** |
| Q5 (0.813 <) | 0.98 (0.92–1.04) | 0.95 (0.89–1.01) |  | 0.99 (0.92–1.06) | 0.95 (0.88–1.02) |  | 0.99 (0.93–1.06) | **0.92 (0.86–0.99)** |
| Cord blood lead |  |  |  |  |  |  |  |  |
| Q1 (< 0.373) | 1 (reference) | 1 (reference) |  | 1 (reference) | 1 (reference) |  | 1 (reference) | 1 (reference) |
| Q2 (0.373–0.451) | 1.00 (0.77–1.29) | 1.04 (0.79–1.36) |  | 1.10 (0.83–1.46) | 1.10 (0.82–1.47) |  | 0.98 (0.76–1.26) | 1.01 (0.77–1.31) |
| Q3 (0.451–0.540) | 0.91 (0.70–1.19) | 0.93 (0.70–1.24) |  | 0.95 (0.71–1.28) | 0.92 (0.68–1.26) |  | 1.01 (0.79–1.29) | 1.02 (0.78–1.32) |
| Q4 (0.540–0.676) | 1.11 (0.86–1.42) | 1.12 (0.86–1.46) |  | 1.16 (0.88–1.54) | 1.07 (0.80–1.44) |  | 1.01 (0.79–1.29) | 1.02 (0.79–1.33) |
| Q5 (0.676 <) | 1.01 (0.78–1.31) | 0.97 (0.73–1.28) |  | 1.19 (0.90–1.57) | 1.07 (0.80–1.44) |  | 0.95 (0.74–1.23) | 0.88 (0.66–1.15) |
| Values are presented as relative risks (99% confidence intervals) adjusted for sex, gestational ages at maternal blood sampling and at birth, birthweight, mother's age, maternal smoking, maternal and paternal education, household income, continuous breastfeeding until 6 months, and home speech stimulation at 1 month. Bold text represents statistical significance (p < 0.01).  *sNDD* suspected neurodevelopmental delay, *Q* quintile | | | | | | | | |

| **Supplementary Table S4.** Sex-stratified association between prenatal lead and sNDD using 99% confidence intervals of lead exposure | | | | | | | | |
| --- | --- | --- | --- | --- | --- | --- | --- | --- |
| **a.** Log-transformed   Lead (log_10_) | sNDD-1Y | |  | sNDD-2Y | |  | sNDD-3Y | |
|  | crude | adjusted |  | crude | adjusted |  | crude | adjusted |
| Maternal blood |  |  |  |  |  |  |  |  |
| Boy | 0.97 (0.82-1.16) | 0.93 (0.78-1.12) |  | 0.94 (0.78-1.12) | 0.88 (0.72-1.06) |  | 0.91 (0.77-1.07) | 0.85 (0.72-1.02) |
| Girl | 0.91 (0.75-1.11) | 0.90 (0.74-1.11) |  | 0.95 (0.75-1.18) | 0.91 (0.72-1.16) |  | 0.88 (0.69-1.13) | 0.82 (0.63-1.07) |
| Cord blood |  |  |  |  |  |  |  |  |
| Boy | 1.08 (0.55-2.08) | 1.06 (0.52-2.12) |  | 1.25 (0.63-2.46) | 1.21 (0.59-2.46) |  | 0.77 (0.43-1.35) | 0.74 (0.41-1.35) |
| Girl | 1.04 (0.49-2.18) | 1.13 (0.51-2.42) |  | 1.60 (0.67-3.75) | 1.53 (0.61-3.78) |  | 1.28 (0.52-3.11) | 1.35 (0.51-3.54) |
|  |  |  |  |  |  |  |  |  |
| **b.** Quintiles of   Lead (µg/dL) | sNDD-1Y | |  | sNDD-2Y | |  | sNDD-3Y | |
|  | crude | adjusted |  | crude | adjusted |  | crude | adjusted |
| Maternal blood |  |  |  |  |  |  |  |  |
| Boy |  |  |  |  |  |  |  |  |
| Q1 (< 0.468) | 1 (reference) | 1 (reference) |  | 1 (reference) | 1 (reference) |  | 1 (reference) | 1 (reference) |
| Q2 (0.468–0.564) | **0.92 (0.84-1.00)** | 0.92 (0.84-1.00) |  | 0.97 (0.89-1.06) | 0.96 (0.87-1.05) |  | 0.96 (0.89-1.04) | 0.95 (0.88-1.03) |
| Q3 (0.564–0.666) | 0.94 (0.86-1.02) | 0.94 (0.86-1.03) |  | 0.94 (0.86-1.02) | 0.93 (0.85-1.02) |  | **0.91 (0.84-0.99)** | **0.90 (0.83-0.98)** |
| Q4 (0.666–0.813) | **0.92 (0.84-1.00)** | 0.92 (0.84-1.01) |  | 0.95 (0.87-1.03) | 0.95 (0.87-1.04) |  | **0.93 (0.86-1.00)** | 0.92 (0.85-1.00) |
| Q5 (0.813 <) | 0.97 (0.90-1.05) | 0.95 (0.87-1.04) |  | 0.97 (0.89-1.06) | 0.94 (0.85-1.03) |  | 0.96 (0.89-1.04) | 0.94 (0.86-1.02) |
| Girl |  |  |  |  |  |  |  |  |
| Q1 (< 0.468) | 1 (reference) | 1 (reference) |  | 1 (reference) | 1 (reference) |  | 1 (reference) | 1 (reference) |
| Q2 (0.468–0.564) | 0.96 (0.87-1.05) | 0.97 (0.88-1.06) |  | 0.92 (0.82-1.02) | 0.94 (0.84-1.06) |  | 0.94 (0.84-1.06) | 0.95 (0.84-1.08) |
| Q3 (0.564–0.666) | 0.93 (0.85-1.02) | 0.97 (0.88-1.07) |  | 0.98 (0.88-1.09) | 1.01 (0.90-1.13) |  | 0.91 (0.81-1.03) | 0.90 (0.80-1.03) |
| Q4 (0.666–0.813) | 0.91 (0.83-1.00) | 0.93 (0.84-1.02) |  | 0.96 (0.86-1.07) | 0.97 (0.86-1.09) |  | 0.92 (0.82-1.04) | 0.95 (0.83-1.07) |
| Q5 (0.813 <) | 0.96 (0.88-1.05) | 0.95 (0.86-1.05) |  | 0.97 (0.87-1.08) | 0.96 (0.85-1.08) |  | 0.95 (0.85-1.07) | 0.90 (0.79-1.02) |
| Cord blood lead |  |  |  |  |  |  |  |  |
| Boy |  |  |  |  |  |  |  |  |
| Q1 (< 0.373) | 1 (reference) | 1 (reference) |  | 1 (reference) | 1 (reference) |  | 1 (reference) | 1 (reference) |
| Q2 (0.373–0.451) | 0.93 (0.65-1.33) | 0.96 (0.66-1.40) |  | 1.02 (0.70-1.47) | 0.98 (0.66-1.44) |  | 0.98 (0.73-1.32) | 1.04 (0.76-1.41) |
| Q3 (0.451–0.540) | 0.92 (0.64-1.31) | 0.94 (0.65-1.37) |  | 0.90 (0.61-1.31) | 0.85 (0.57-1.27) |  | 1.02 (0.76-1.35) | 1.03 (0.76-1.41) |
| Q4 (0.540–0.676) | 1.14 (0.82-1.58) | 1.15 (0.81-1.63) |  | 1.16 (0.82-1.64) | 1.09 (0.76-1.58) |  | 0.91 (0.68-1.22) | 0.98 (0.72-1.33) |
| Q5 (0.676 <) | 0.93 (0.66-1.32) | 0.89 (0.61-1.30) |  | 1.11 (0.78-1.58) | 1.04 (0.72-1.52) |  | 0.92 (0.69-1.23) | 0.88 (0.64-1.21) |
| Girl |  |  |  |  |  |  |  |  |
| Q1 (< 0.373) | 1 (reference) | 1 (reference) |  | 1 (reference) | 1 (reference) |  | 1 (reference) | 1 (reference) |
| Q2 (0.373–0.451) | 1.08 (0.74-1.58) | 1.13 (0.76-1.68) |  | 1.23 (0.79-1.93) | 1.28 (0.81-2.02) |  | 1.00 (0.63-1.57) | 0.98 (0.59-1.60) |
| Q3 (0.451–0.540) | 0.90 (0.60-1.35) | 0.92 (0.60-1.40) |  | 1.04 (0.65-1.67) | 1.03 (0.63-1.69) |  | 0.98 (0.62-1.56) | 0.98 (0.60-1.60) |
| Q4 (0.540–0.676) | 1.04 (0.70-1.54) | 1.05 (0.70-1.60) |  | 1.13 (0.70-1.80) | 1.03 (0.62-1.71) |  | 1.14 (0.73-1.79) | 1.09 (0.68-1.75) |
| Q5 (0.676 <) | 1.11 (0.75-1.62) | 1.12 (0.74-1.69) |  | 1.29 (0.82-2.03) | 1.12 (0.69-1.82) |  | 0.95 (0.59-1.52) | 0.89 (0.52-1.50) |
| Values are presented as relative risks (99% confidence intervals) adjusted for sex, gestational ages at maternal blood sampling and at birth, birthweight, mother's age, maternal smoking, maternal and paternal education, household income, continuous breastfeeding until 6 months, and home speech stimulation at 1 month. Bold text represents statistical significance (p < 0.01).  *sNDD* suspected neurodevelopmental delay, *Q* quintile | | | | | | | | |
